# Sensitive, Selective and Reliable Detection of Fe^3+^ in Lake Water via Carbon Dots-Based Fluorescence Assay

**DOI:** 10.3390/molecules27196749

**Published:** 2022-10-10

**Authors:** Zhuang Xiang, Yuxiang Jiang, Chen Cui, Yuanping Luo, Zhili Peng

**Affiliations:** 1 School of Materials and Energy, Yunnan University, Kunming 650091, China; 2 Yunnan Key Laboratory for Micro/Nano Materials & Technology, Yunnan University, Kunming 650091, China

**Keywords:** Fe^3+^, fluorescence sensing, carbon dots, lake water, limit of detection

## Abstract

In this study, C-dots were facilely synthesized via microwave irradiation using citric acid and ethylenediamine as carbon precursors. The fluorescence emissions of the C-dots could be selectively quenched by Fe^3+^, and the degree of quenching was linearly related to the concentrations of Fe^3+^ presented. This phenomenon was utilized to develop a sensitive fluorescence assay for Fe^3+^ detection with broad linear range (0–250, 250–1200 μmol/L) and low detection limit (1.68 μmol/L). Most importantly, the assay demonstrated high reliability towards samples in deionized water, tap water and lake water, which should find potential applications for Fe^3+^ monitoring in complicated environments.

## 1. Introduction

Ferric ions (Fe^3+^) play essential roles and could participate in many important biological metabolic processes, such as RNA and DNA synthesis, oxygen transport, electron transfer and the formation of heme in the human body [1,2,3]. As such, proper intake of Fe^3+^ is of great importance for human health; too much Fe^3+^ consumed over a long period of time could cause harm to the human body. Studies have shown that continuous consumption of unbalanced Fe^3+^ might be linked to a range of conditions including Parkinson’s disease, Alzheimer’s disease and anemia [4,5,6]. As is well known, most of Fe^3+^ for the human body mainly comes from food and water; thus, it is of great significance to monitor Fe^3+^ content in drinking water. As a matter of fact, the European Union requires that the maximum permissible concentration of Fe^3+^ in drinking water is 3.57 μmol/L.

Compared with the traditional colorimetric method [7], atomic absorption spectrometry [8], and inductively coupled plasma mass spectrometry [9], fluorescence sensing is a relatively new detection method, which has the advantages of high sensitivity, easy operation and good reproducibility [10,11]. As such, many fluorescence sensing platforms have been developed for the sensitive and selective detection of Fe^3+^ [12], among which carbon dots (C-dots)-based sensing platforms have received enormous attention [10,13,14]. As a new type of carbon-based fluorescent nanomaterials, C-dots have superior properties such as excellent photoluminescence (PL) [15], high biocompatibility [16,17], facile surface functionalization [18,19] as well as low toxicity [20,21] and cost [22], which are ideal for fluorescence sensing assays development [23].

In the past few years, various C-dots-derived sensing platforms have been developed for the detection of Fe^3+^ (Table S1), which greatly enriched the toolbox for Fe^3+^ detection and monitoring. Still, these Fe^3+^ sensing assays generally face some challenges, such as impractical limit of detection (LOD) [24,25,26,27,28,29], in which the LODs of the assays were above the maximum permissible concentration of Fe^3+^ in drinking water (3.57 μmol/L) as set by the European Union. On the other hand, some assays had excellent LODs; however, their linear ranges were very narrow or biased [30,31], which significantly limited their practical applications. Furthermore, although some of the assays might have relatively good LODs and balanced linear ranges towards Fe^3+^, their applications in noncontrolled water samples (i.e., natural water) have not been demonstrated [32,33,34,35]. Assays that have excellent LODs, balanced linear ranges and have been demonstrated for applications in noncontrolled water samples are relatively scarce [36,37]. As such, it is still of great necessity and importance to develop C-dots-derived sensing assays capable of the sensitive and selective detection of Fe^3+^ in natural water samples.

Herein, we report the development of C-dots-based sensing assays for the sensitive and selective detection of Fe^3+^. Specifically, the C-dots used in this study were facilely prepared from citric acid and ethylenediamine using a microwave synthesizer. PL of aqueous dispersion of the C-dots could be efficiently quenched by Fe^3+^ solution, and the degree of quenching is linearly related to the concentration of Fe^3+^ solution. Exploiting this correlation, a facile sensing assay was developed with broad linear ranges (0–250, 250–1200 μmol/L) and a LOD of 1.68 μmol/L; the assay also demonstrated high selectivity towards Fe^3+^, free from the interference of 14 common ions. Furthermore, the assay was found to be accurate for deionized (DI) water samples, tap water samples and natural lake water samples, indicating the high practicality of this assay.

## 2. Results and Discussion

### 2.1. Characterizations of C-Dots

**Figure 1:**
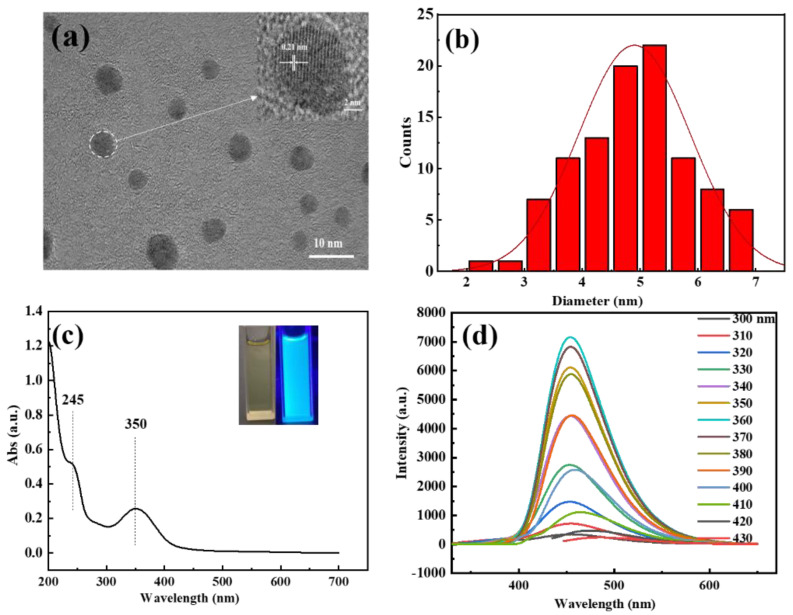
(**a**) TEM image of C-dots showing the high-resolution lattice fringes (insert); (**b**) the size distribution histogram; (**c**) the UV-Vis spectrum of C-dots and the dispersion of C-dots under UV-light excitation (inset, left) and under ambient light (inset, right); (**d**) the fluorescence emissions of C-dots (8.3 mg/L in DI water) under the excitation of lights with different wavelengths, as indicated in the figure.

The as-prepared C-dots were brown gels, which were typical for citric acid and ethylenediamine-derived C-dots [38]. The C-dots were fully characterized by TEM, UV-Vis, fluorescence, FTIR, XPS and XRD to investigate their morphological, optical, chemical and structural properties. TEM was applied first to explore the morphological and structural characteristics of the sample. As can be seen, the C-dots were well-dispersed spherical particles with no agglomerates (Figure 1a), the diameters of these particles range from 2.3 to 6.8 nm, with an average diameter of 4.9 nm (Figure 1b). Furthermore, well-resolved lattice fringes with an interplanar spacing of 0.21 nm could be clearly observed in the HRTEM (Figure 1a, inset), which could be attributed to the (100) facet of graphite, indicating the successful carbonization and graphitization of the carbon precursors.

Next, we studied the spectroscopic behaviors of the C-dots with UV-Vis and fluorescence spectroscopy. The aqueous dispersion of the C-dots appeared pale yellow under ambient light and turned bright blue upon being excited by UV light at 365 nm (Figure 1c, inset). The UV-Vis absorption spectrum of C-dots demonstrated two main absorption peaks at 245 and 350 nm in the UV region (Figure 1c), which could be attributed to the n–σ* transition of -NH_2_ and the n–π* transition of C=O [39,40], respectively. Interestingly, unlike most of the reported C-dots that had excitation-wavelength-dependent emissions, the sample presented strong fluorescence emissions that were independent of the excitation wavelength, with the optimal emission and excitation wavelengths at 453 and 360 nm, respectively (Figure 1d). The absolute fluorescence quantum yield of the C-dots was determined to be 40.87% when the sample was dispersed in DI water with a concentration of 66.4 mg/L.

**Figure 2:**
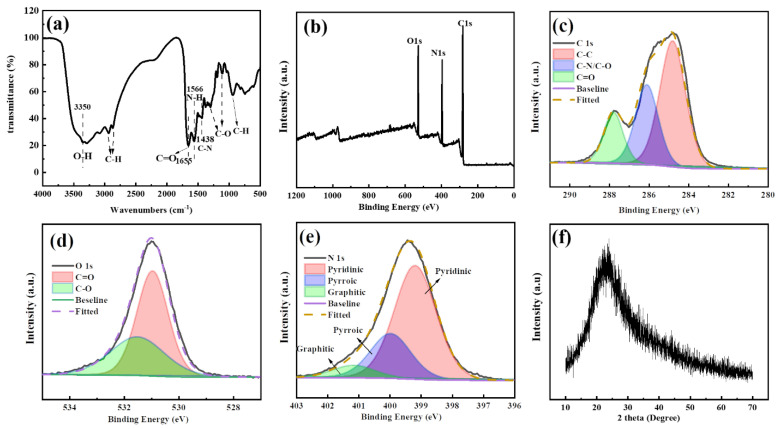
(**a**) The FTIR spectrum of C-dots; (**b**) full-scale XPS spectrum of C-dots; (**c**) high-resolution XPS spectra of C 1s; (**d**) high-resolution XPS spectra of O1s; (**e**) high-resolution XPS spectra of N 1s; (**f**) XRD pattern of C-dots.

FTIR was then performed to analyze the surface functional groups of the C-dots (Figure 2a). As can be seen, the peak at 3350 cm^−1^ could be attributed to the stretching vibration of O-H, and peaks at 2938, 2872 and 950 cm^−1^ could be ascribed to the stretching vibrations of C-H [27]. The peaks at 1655, 1566, and 1438 cm^−1^ could be assigned to the bend vibrations from C=O [41], N-H and C-N, respectively, indicating the presence of amide bonds in the samples. Furthermore, the peaks at 1376 and 1041 cm^−1^ could be attributed to the stretching vibrations of the C-O bond [30,42]. Collectively speaking, the FTIR analysis indicated that there were hydroxyls, amide and alkyl groups on the surface of C-dots, which were expected since the C-dots were prepared from citric acid and ethylenediamine. The results also corresponded well with the analysis from the UV-Vis spectrum in which absorption peaks originated from C-NH_2_ and C=O bonds were observed.

To further explore the surface chemical states of the C-dots, the XPS spectroscopy of the sample was also investigated. Clearly, the full-scan XPS spectrum of C-dots exhibited three main peaks at 529.8, 396.8, and 284.6 eV, corresponding to O1s, N1s, and C1s, respectively (Figure 2b). Based on the XPS spectrum, the C-dots were mainly composed of carbon, nitrogen and oxygen, with a percentage of 66.16, 19.73 and 14.05%, respectively, indicating that the C-dots have been sufficiently carbonized and nitrogen-doped. As shown, the high-resolution C1s spectrum revealed three main peaks at 284.80, 286.18 and 287.79 eV, which were assigned to C-C/C=C, C-N/C-O and C=O, respectively (Figure 2c). Similarly, the high-resolution spectrum of O1s demonstrated two peaks at 530.94 and 531.99 eV, which could be attributed to C=O and C-O, respectively (Figure 2d). The high-resolution spectrum of N1s had three peaks at 399.16, 400 and 401.19 eV corresponding to pyridinic, pyrrolic and graphitic nitrogen [43], respectively (Figure 2e). In summary, the XPS analysis indicated that there was a rich presence of C=C, C=O, C-O and C-N on the surface of the C-dots, which corresponded well with the analysis from FTIR and UV-Vis spectroscopy.

To further study the structural features of the C-dots, XRD analysis was performed on the C-dots (Figure 2f). As can be seen, the XRD pattern of the C-dots demonstrated a broad peak centered around 24°, which was typical for C-dots and generally indicated the successful carbonization and synthesis of C-dots [44]. Moreover, the peak could be attributed to the (002) facet with an interlayer spacing d of 0.37 nm [45], which is larger than the typical interlayer spacing of 0.34 nm in graphite. The increased d value indicated that there was an increase in the amorphous nature of the C-dots, which could be attributed to the introduction of the oxygen-containing functional groups [46].

### 2.2. Sensing Assay Development

**Figure 3:**
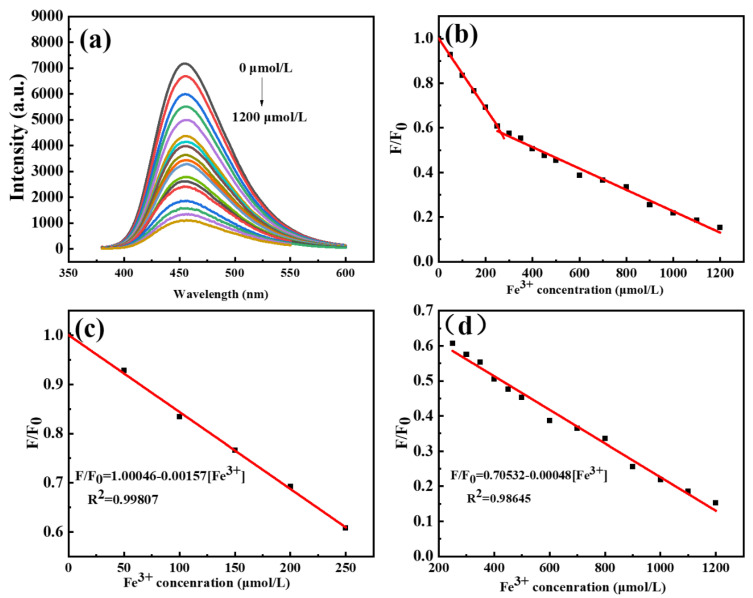
(**a**) Fluorescence emission spectra of C-dots in the presence of Fe^3+^ ions of various concentrations ranging from 0 to 1200 μmol/L; the emissions were excited at the optimal excitation wavelength of 360 nm and recorded at 453 nm; (**b**) scheme showing the correlation between F/F_0_ (y-axis) and concentrations of Fe^3+^ (x-axis) where F_0_ and F represented the emission intensities of the blank C-dots dispersions and C-dots dispersions with Fe^3+^ presented, respectively; (**c**) linear detection range for Fe^3+^ from 0 to 250 μmol/L; (**d**) linear detection range for Fe^3+^ from 250 to 1200 μmol/L).

With the full characterization of C-dots, we set our step to develop a sensitive assay using C-dots as the platform. To our delight, we found that Fe^3+^ could efficiently quench the PL intensity of C-dots, and the degree of quenching was closely correlated to the concentrations of Fe^3+^, making it possible to develop a sensitive assay for the detection of Fe^3+^. Indeed, as the concentrations of Fe^3+^ gradually increased from 0 to 1200 μmol/L, the emission intensities of C-dots dispersions at 453 nm decreased accordingly (Figure 3a). To elucidate the relationship between the concentrations of Fe^3+^ and the PL intensities of C-dots, a scheme where F/F_0_ represented the y-axis and concentrations of Fe^3+^ stood for the x-axis was plotted (Figure 3b), where F_0_ and F represented the emission intensities of the blank C-dots dispersions and C-dots dispersions with Fe^3+^ presented, respectively. As can be seen, the PL intensities of C-dots are linearly related to the concentrations of Fe^3+^ in two ranges; one is from 0 to 250 μmol/L and the other one is from 250 to 1200 μmol/L (Figure 3b). As demonstrated in the calibration curves, both the two ranges have very good linear relationships between Fe^3+^ concentrations and the intensities of C-dots dispersions (F/F_0_). Specifically, the first fitting curve (0–250 μmol/L) has a linear equation of F/F_0_ = 1.00046–0.00157 [Fe^3+^] with R^2^ = 0.99807 (Figure 3c), and the second fitting curve (250–1200 μmol/L) has a linear equation of F/F_0_= 0.70532–0.00048 [Fe^3+^] with R^2^ = 0.99371 (Figure 3d). According to the well-accepted 3σ method, LOD = 3 σ/k, where σ is the standard deviation of the blank samples and k is the slope of the linear calibration plot [47,48,49]. The LOD for the assay based on the calibration curve presented in Figure 3c was determined to be 1.68 μmol/L, which is well below the maximum permissible concentration of Fe^3+^ in drinking water (3.57 μmol/L), as required by the European Union. In summary, the sensing assay developed in this study has a broad linear range and a very low LOD.

### 2.3. Selectivity and Reliability of the Assay

**Figure 4:**
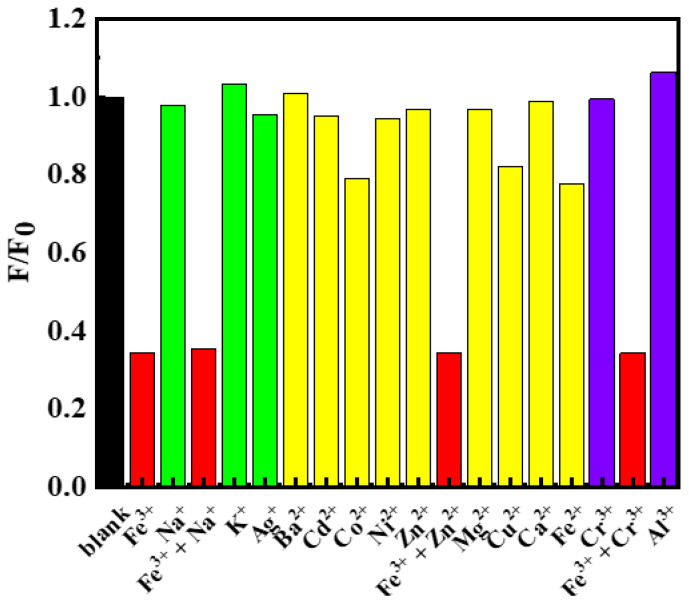
Fluorescence emission intensity changes of C-dots dispersions in the presence of 750 μmol/L of different metal ions including Fe^3+^, Na^+^, (Fe^3+^ + Na^+^), K^+^, Ag^+^, Ba^2+^, Cd^2+^, Co^2+^, Ni^2+^, Zn^2+^, (Fe^3+^ + Zn^2+^), Mg^2+^, Cu^2+^, Ca^2+^, Fe^2+^, Cr^3+^, (Fe^3+^ + Cr^3+^) and Al^3+^. F_0_ represented the emission intensity of the blank C-dots dispersion and F represented the emission intensities of the C-dots dispersions in the presence of the respective metal ions.

With the successful establishment of the sensing assay, we also carried out careful experiments to investigate the selectivity of the assay. As known, many ions often co-occurred in solutions, thus the ability to be free from the interference of other ions is very important for a metal-ion sensing assay. To our delight, the assay developed was free from most of the commonly seen metal ions. As can be seen, common monovalent ions such as Na^+^, K^+^ as well as Ag^+^ hardly alter the PL intensity of C-dots dispersions (Figure 4, green columns), and thus could not interfere with the detection of Fe^3+^. Similarly, nine commonly seen divalent metal ions, namely, Ba^2+^, Cd^2+^, Co^2+^, Ni^2+^, Zn^2+^, Mg^2+^, Cu^2+^, Ca^2+^ and Fe^2+^, were also tested, which also had no serious interference to the detection of Fe^3+^ (Figure 4, yellow columns). It is worth noting that, as Fe^2+^ had no significant interference to Fe^3+^, the current assay has the potential to be developed as a Fe^2+^ sensing assay by adding oxidants into the solutions in advance of testing [50]. Lastly, we also tested common trivalent metal ions such as Cr^3+^ and Al^3+^; they also had no interference to the detection of Fe^3+^ (Figure 4, purple columns). Encouraged by these findings, we further evaluated the selectivity of Fe^3+^ detection in the presence of second cations including Na^+^, Zn^2+^ and Cr^3+^. The results showed that the co-presence of these cations did not have obvious interferences on the sensing of Fe^3+^ (Figure 4, red columns). In summary, the sensing assay developed in this study had excellent selectivity towards Fe^3+^ and was free from the interference of most metal ions.

To test the reliability of the sensing assay developed, four spike solutions of Fe^3+^ in DI water were prepared and their concentrations were determined using the above-established calibrations curves, which were then compared with the actual concentrations. To our delight, the assay developed in this study seemed very reliable towards all the spike solutions tested (Table 1, rows 1–4). Specifically, the recoveries of the four spike samples ranged from 92.85 to 105.50%, which were all within the tolerance range for a sensing assay. Furthermore, extremely high recovery of 99.58% could be achieved when the concentration of Fe^3+^ solution was set at 50 μmol/L (Table 1, row 1).

As all the studies above were carried out with laboratory-made DI water, to explore the practicality of our assay for the detection of Fe^3+^ in other water samples, additional experiments were conducted. Specifically, we collected tap and lake water and the presence of Fe^3+^ in these two samples were investigated. As demonstrated, the fluorescence intensity of C-dots in DI water was identical to that of C-dots in tap and lake water (Figure S1), indicating that the presence of Fe^3+^ in these two samples was well below the LOD of this assay. Thus, to better evaluate the reliability of this assay in non-laboratory water (i.e., tap and lake water), we made seven spike solutions of Fe^3+^, three with tap water and four with natural lake water. To our surprise, the assay was also very reliable towards the spike solutions made from the everyday water samples. Specifically, the recoveries of the three tap water samples ranged from 92.67 to 99.01% (Table 1, rows 5–7), while the four lake water samples ranged from 93.52 to 99.45% (Table 1, rows 8–11), all of which had very excellent recovery percentages. Similarly, some extremely high recovery yields were also observed for both tap water samples (99.01%, Table 1, row 5) and lake water samples (99.45%, Table 1, row 11), demonstrating the high practicality of the assay reported in this study.

In conclusion, compared to the literature precedents [24,25,26,27,28,29] listed in Table S1, the assay developed in this study had a relatively good LOD that meets the European Union standards for the detection of drinking water. In addition, the assay also had a wide linear range that was better than most of the reported assays [24,25,26,27,28,29,30,31,32,33,34,36,37], and should be sufficient for most everyday sensing needs. Most importantly, we have demonstrated the potential application of this assay for Fe^3+^ sensing in natural waters, and the results were better or close to the reported work [36,37].

### 2.4. Possible PL Quenching Mechanisms

The quenching of a fluorophore by quenchers generally occurred via dynamic quenching (collisional inactivation) or static quenching (static complexation). In a typical static quenching, a ground-state complex is formed through the interaction between C-dots and the quencher. The complex is generally nonfluorescent and could return to the ground state without PL emissions upon absorbing a light [51]. For static quenching, the fluorescence lifetime of C-dots should stay unchanged with or without a quencher, that means τ_0_/τ = 1. Furthermore, the formation of a non-fluorescent complex generally should result in new peaks in the C-dots absorption spectrum.

**Figure 5:**
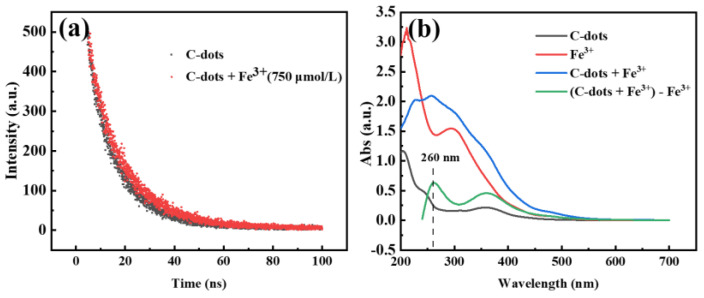
(**a**) The fluorescence decay curves of C-dots in the absence (black) and presence of Fe^3+^ ions (red); (**b**) UV-Vis absorption spectra of C-dots only (black), Fe^3+^ solution (red), C-dots in the presence of Fe^3+^ (blue) and the mathematical curve (green) resulted by subtracting the red curve (Fe^3+^ only) from the blue curve (C-dots + Fe^3+^), respectively. The negative portion of spectrum in the mathematically calculated green curve was not shown, as it has no physical meaning.

To explore the quenching mechanism, Time-correlated single-photon counting (TCSPC) experiments were carried out to investigate the charge transfer and exciton recombination processes of C-dots in the presence and absence of Fe^3+^ ions. Based on the tests, the fluorescence lifetime of C-dots without Fe^3+^ was determined to be 11.2 ns, while that in the presence of Fe^3+^ was determined to be 12 ns (Figure 5a). These results indicated that the presence of Fe^3+^ did have an influence on the excited states of C-dots; however, the influence was quite minimal. We then carefully evaluated the complexation of C-dots with Fe^3+^ using UV-Vis absorption spectroscopy (Figure 5b). It was clear that a new absorption peak at 260 nm appeared for C-dots in the presence of Fe^3+^ (green curve vs. black curve), which might be attributed to the complexation of C-dots with Fe^3+^, and this is generally considered to be a sign of static quenching [52,53,54]. According to these results, we cautiously propose that the quenching of the fluorescence of C-dots by Fe^3+^ was mainly via the static quenching path.

## 3. Materials and Methods

### 3.1. Reagents and Materials

KCl, NaCl, AgNO_3_, CuSO_4_, CaCl_2_, MgCl_2_, CoCl_2_·6H_2_O, NiCl_2_, Zn(CH_3_COO)_2_, BaCl_2_, CdCl_2_, FeCl_2_, Pb(NO_3_)_2_, FeCl_3_, AlCl_3_, MnCl_2_·4H_2_O and CrCl_3_·6H_2_O were purchased from Energy Chemical Reagent Co., Ltd. (Shanghai, China). Citric acid and anhydrous ferric chloride (FeCl_3_) were obtained from Sahn Chemical Technology Co., Ltd. (Shanghai, China). Ethylene diamine was purchased from Shanghai Aladdin Biochemical Technology Co., Ltd. (Shanghai, China). The deionized water used in all experiments was made from a Master Touch-S laboratory ultrapure water machine (Master Touch, Shanghai, China). Lake water was taken from the Ze Lake at the Chenggong Campus of Yunnan University, Kunming, Yunnan, China. All the reagents were used as received without further purification, unless otherwise noted.

### 3.2. Synthesis of C-Dots

The C-dots used in this study were prepared as follows: briefly, a mixture of citric acid (4 g) and ethylenediamine (20 mL) was heated in a microwave synthesizer set at 160 °C for 10 min. The resulted reaction mixture was then evaporated at 80 °C for 4 h in a rotary evaporator to remove the excess ethylenediamine, which resulted in the gelatinous brown C-dots.

### 3.3. Characterizations of C-Dots

The UV-Vis absorption spectrum of C-dots was tested on a UV-Vis spectrophotometer (UV-2600, Shimadzu, Tokyo, Japan), in which the wavelength range was set at 195–1100 nm, the scanning speed was set at medium speed, the sampling interval was set at 1 nm, and all the samplings were repeated twice. The concentration of the C-dots dispersion used for the test was 33.2 mg/L. The fluorescence spectrum of C-dots (8.3 mg/L) was tested by a fluorescence spectrometer (FL, F97 Pro, Shanghai Prism Technology Co., Ltd., Shanghai, China), in which a three-dimensional wavelength scanning mode was adopted. The excitation and emission wavelengths were set at 220–500, and 220–700 nm, respectively; both the excitation and emission widths were set at 10 nm. The scanning speed was set at 1000 nm/min, the scanning interval was set at 1 nm and the gain was set at 650 V. The frequency-domain lifetime was measured by a FLS10000 fluorescence lifetime spectrometer (Edinburgh, UK).

The Fourier transform infrared (FTIR) spectra were measured using a FTIR spectrometer (Nicoletis10, Thermo Scientific, Waltham, MA, USA), in which samples were smeared on KBr before testing. The elemental composition of C-dots was analyzed by a multifunctional X-ray photoelectron spectroscopy (XPS, K-Alpha, Thermo, USA).

The transmission electron microscopy (TEM) of C-dots was measured on a transmission electron microscope (JEM-2100, JEOL, Tokyo, Japan), in which the acceleration voltage was set at 200 kV, and the magnification was 300,000 times. The samples for TEM testing were treated as follows: 50 μL of C-dots dispersion (0.5 g/L) were dispersed in 10 mL of water, and then sonicated for 30 min. Then, a drop of C-dots dispersion was taken and placed on carbon net until the moisture was volatilized before they were applied for the TEM test. The X-ray diffraction (XRD) pattern was obtained on an X-ray diffractometer (DX-2700BH, Haoyuan Instrument Co., Ltd., Dandong, Liaoning, China) with a wavelength (λ) of 0.15406 nm.

### 3.4. Sensing Assay Development

Detection of Fe^3+^ in aqueous solutions: Firstly, mother solution/dispersion of Fe^3+^ and C-dots with a concentration of 3 mmol/L and 500 mg/L, respectively, were prepared. Then, the two solutions/dispersions were mixed accordingly to generate a series of mix dispersions with the concentrations for C-dots as constant (8.3 mg/L), and the concentrations for Fe^3+^ as gradients (0, 50, 100, 150, 200, 250, 300, 350, 400, 450, 500, 600, 700, 800, 900, 1000, 1100, and 1200 μmol/L). The mix dispersions were allowed to set for 5 min before their PL emissions were measured.

Selectivity of the assay: All the procedures were similar to the above discussions except that the concentrations of the metal ions (Fe^3+^, Na^+^, K^+^, Ag^+^, Ba^2+^, Cd^2+^, Co^2+^, Ni^2+^, Zn^2+^, Mg^2+^, Cu^2+^, Ca^2+^, Fe^2+^, Cr^3+^, Al^3+^) were set as a constant (750 μmol/L).

Reliability of the assay: To test the reliability of the assay, 11 spike solutions of Fe^3+^ in DI water, tap water and lake water were prepared, and their concentrations were determined based on the calibration curves established in this study. The determined concentrations of Fe^3+^ were then compared with their actual concentrations to calculate the recovery percentage.

## 4. Conclusions

A facile synthesis of C-dots via microwave irradiation, with citric acid as a carbon precursor and ethylenediamine as a passivation agent, was developed. The C-dots demonstrated excitation-wavelength-independent emissions at 453 nm, and the emissions could be effectively quenched by Fe^3+^. Exploiting this phenomenon, a sensitive assay for the detection of Fe^3+^ in its aqueous solutions was developed, which achieved broad linear ranges (0–250, 250–1200 μmol/L) and excellent LOD (1.68 μmol/L). Furthermore, the assay also demonstrated excellent selectivity towards Fe^3+^ and was free from interference of most commonly seen metal ions. Most importantly, the assay developed in this study functioned well with high reliability towards both DI water and everyday water (i.e., tap water and lake water), demonstrating its high practicality towards Fe^3+^ in environmental water samples.

## Figures and Tables

**Table 1:** Sensing reliability of the assay developed as demonstrated with Fe^3+^ spike solutions in DI water, tap water and lake water.

	Sample	Fe^3+^ Added (μmol/L)	Fe^3+^ Found (μmol/L)	Recovery (%)
1	DI water	50	49.79	99.58
2	DI water	100	105.50	105.50
3	DI water	150	139.27	92.85
4	DI water	200	196.32	98.16
5	Tap water	100	100.99	99.01
6	Tap water	150	161.06	92.67
7	Tap water	200	208.17	95.92
8	Lake water	50	47.93	95.86
9	Lake water	100	100.93	99.07
10	Lake water	150	159.72	93.52
11	Lake water	200	198.90	99.45

## Data Availability

The data presented in this study are available within the article and supplementary material).

## Supplementary Materials

The following supporting information can be downloaded at: https://www.mdpi.com/article/10.3390/molecules27196749/s1, Table S1: A summary of the representative reports in which C-dots based fluorescence assays were applied for Fe^3+^ detection; Figure S1: The normalized fluorescence intensities of C-dots dispersions in DI water (green column), tap water (blue column) and lake water (turquoise column), respectively.

## Sample Availability

Samples of the C-dots are available from the authors.
