# Supplementary material for: Sensitive, Selective and Reliable Detection of Fe3+ in Lake Water via Carbon Dots-Based Fluorescence Assay
Source: Molecules. 2022 Oct 10;27(19):6749. doi: 10.3390/molecules27196749 (PMC9573028; doi:10.3390/molecules27196749)
Supplement: Supplementary file 1 [file molecules-27-06749-s001.zip › molecules-1954704-supplementary.pdf]

Supplementary Materials

Sensitive, selective and reliable detection of Fe^{3+} in lake water *via* carbon dots-based fluorescence assay

Zhuang Xiang [a,b], Yuxiang Jiang [a], Chen Cui [a], Yuanping Luo [a] and Zhili Peng [a,*]

[a]School of Materials and Energy, Yunnan University, Kunming 650091, China.

[b]Yunnan Key Laboratory for Micro/Nano Materials & Technology, Yunnan University, Kunming 650091, China.

***Corresponding authors:**

(Z. P.) Tel.: +86–871–65037399; E–mail: zhilip@ynu.edu.cn.

Keywords: Fe^{3+}; fluorescence sensing; carbon dots; lake water; limit of detection

Table S1. A summary of the representative reports in which C-dots based fluorescence assays were applied for Fe^{3+} detection

	Carbon precursor[a)]	LOD (μmol/L)	Linear range (μmol/L)	Natural water sample testing	Ref[b)].
**1**	Folic acid and NAAMDS	30	100-1000	-	[24]
**2**	CA and DHP	20	20-200	-	[25]
**3**	Alginic acid and EDA	10.98	0−50	Yes	[26]
**4**	Cranberry beans	9.55	30-600	Yes	[27]
**5**	L-Glutamic acid	4.67	0-50	-	[28]
**6**	A-lipoic acid	4	25-500	-	[29]
**7**	CA and EDA	2.37	1600-6000	-	[30]
**8**	M-aminobenzoic acid	0.05	0-1.6	Yes	[31]
**9**	L-lactic acid and EDA	1.89	0-200	-	[32]
**10**	DL-malic acid, EDA and EA	0.8	6-200	-	[33]
**11**	Snake gourd peels	0.398	10-100	-	[34]
**12**	Orange peel	0.25	0.5-1000	-	[35]
**13**	Dopamine	2.86	5-200	Yes	[36]
**14**	Trisodium citrate and chicken blood	0.23	0-100	Yes	[37]
**15**	CA and EDA	1.68	0-250,250-1200	Yes	**This work**

[a)]NAAMDS:N-(β-aminoethyl)-γ-aminopropyl methyl dimethoxy silane; CA: citric acid; DHP: diammonium hydrogen phosphate; EDA: ethylenediamine; EA: ethane-sulfonic acid;

[b)] The number of the references in the table is in consistent with the main text.

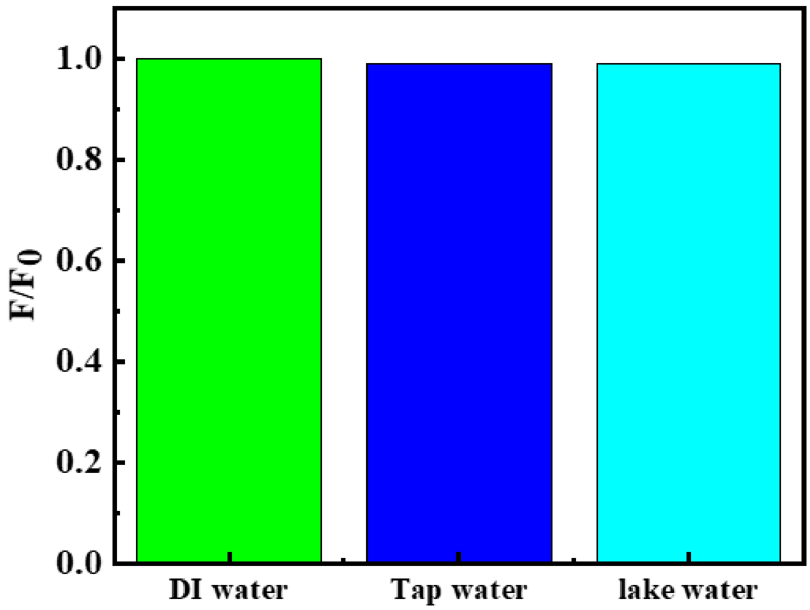

Figure S1 The normalized fluorescence intensities of C-dots dispersions in DI water (green column), tap water (blue column) and lake water (turquoise column), respectively.
